# New Mode of Administering the Sulphate of Quinine

**Published:** 1846-10

**Authors:** 


6. *New Mode of Administering the Sulphate of Quinine.*—A memoir on the use of sulphate of quinine in intermittent fever by friction on the mucous surface of the mouth and fauces, has been addressed to the Academie des Sciences, Paris, by M. Ducros. The following are the conclusions at which he has arrived.—

1. The sulphate of quinine administered in sulphuric ether, by frictions on the tongue, the velum pendulum, the inside of the cheeks, and back of the pharynx, causes an abundant salivation with a strongly marked bitter taste, in the dose of five centigrammes.† The reaction on the spinal marrow excited by this dose is stronger than would have been produced by two grammes‡ taken into the stomach or intestinal canal.

2. The action of sulphate of quinine, administered in this manner, is almost instantaneous, whether employed in malignant intermittents, or in simple agues, or in temporo-facial neuralgia.

3. This immediate therapeutic action is especially important in malignant intermittent, since given in other methods the sulphate of quinine requires to be taken several hours before the paroxysm, while by this method it is sufficient if it be administered half an hour before the access.

4. A still greater advantage of thus employing the quinine in small doses, is the avoiding of all risk of poisoning by the remedy.

5. The rapidity of this action of the quinine in temporo-facial neuralgia is also a most important advantage.—*New York Journal of Medicine.*

